# COVID-19 among Patients Visiting the Department of Emergency of a Tertiary Care Centre: A Descriptive Cross-sectional Study

**DOI:** 10.31729/jnma.8053

**Published:** 2023-05-31

**Authors:** Mahendra Raj Shrestha, Ajaya Basnet, Rajendra Maharjan, Arun Bahadur Chand, Lochan Karki, Subash Singh, Sagar Ghimire, Rupak Maharjan

**Affiliations:** 1 Department of Clinical Laboratory, Nepal Armed Police Force Hospital, Balambu, Kathmandu, Nepal; 2 Department of Medical Microbiology, Shi-Gan International College of Science and Technology, Maharajgani, Kathmandu, Nepal; 3 Department of Clinical Laboratory, KIST Medical College and Teaching Hospital, Mahalaxmi, Lalitpur, Nepal; 4 Department of Medicine, National Academy of Medical Sciences, Bir Hospital, Mahaboudha, Kathmandu, Nepal; 5 Department of Molecular Laboratory, Nepal Armed Police Force Hospital, Balambu, Kathmandu, Nepal; 6 Department of Dermatology, Nepal Armed Police Force Hospital, Balambu, Kathmandu, Nepal.

**Keywords:** *COVID-19*, *Nepal*, *prevalence*, *SARS-CoV-2*

## Abstract

**Introduction:**

Because of the unbridled transmissibility of the SARS-CoV-2 worldwide, researchers and healthcare professionals have set a common goal for timely diagnosis and future prevention of the disease. The aim of this study was to find out the prevalence of COVID-19 among patients visiting the Department of Emergency of a tertiary care centre.

**Methods:**

This descriptive cross-sectional study was conducted among the individuals suspected COVID-19 who had visited the Department of Emergency of a tertiary care centre between 11 January 2021 and 29 December 2021. Ethical approval was taken from Ethical Review Board (Reference number: 2768). Socio-demographic details, clinical symptoms, and two nasopharyngeal swab samples (one in viral transport medium to run RT-PCR and the other for Ag-RDT) were collected from each individual. Convenience sampling method was used. Point estimate and 95% Confidence Interval were calculated.

**Results:**

Among the 232 patients, COVID-19 was detected in 108 (46.55%) (40.13-52.97, 95% CI) by Ag-RDT. A total of 44 (39.63%) of age groups 31-40 years were predominantly infected with SARS-CoV-2. The mean age was 32.13±10.80 years and was mostly males 73 (65.77%). Fever was present in 57 (51.35%) and dry cough was present in 50 (45.05%) COVID-19 patients.

**Conclusions:**

The prevalence of COVID-19 among hospitalized individuals in this study was higher than in previous studies conducted in similar settings.

## INTRODUCTION

As of 25^th^ June 2022, 5,749,355 reverse transcriptase-polymerase chain reaction (RT-PCR) tests have been performed in Nepal and among them, 979,489 tests have diagnosed coronavirus disease of 2019 (COVID-19).^1^

RT-PCR assay although deemed to be the most sensitive and reliable gold standard tool to diagnose SARS-CoV-2 infection in individuals as mentioned by the WHO, is a package of complex laboratory procedures, involving increased cost, prolonged report time, and the need for sophisticated laboratory setup.^2,3^ The immunological diagnosis of COVID-19 by SARS-CoV-2 antigen-based rapid diagnostic test (Ag-RDT) can be the economic and swift alternative to chain the disease. While some international studies have reported high percentages of sensitivity (92.80-98.3%) and specificity (100%) of SARS-CoV-2 Ag-RDT,^4^ several studies have reported lower sensitivity (57.6-81.8%) and specificity (98.7-99.5%) too.^5,6^ However, only a handful of such studies have been published in Nepal.

The aim of this study was to find out the prevalence of COVID-19 among patients visiting the Department of Emergency of a tertiary care centre.

## METHODS

A descriptive cross-sectional study was conducted among COVID-19 suspected symptomatic and asymptomatic individuals, irrespective of age and gender, who had visited the Department of Emergency Services of Nepal Armed Police Force Hospital (NAPFH), Balambu, Kathmandu, Nepal, between 11 January 2021 and 29 December 2021. Ethical approval was taken from the Ethical Review Board (Reference number: 2768). Written informed consent either from study participants (or their relatives) was obtained.

Those individuals, who were recent foreign returnees, or had a travel history to local endemic areas within the past 7 days and who presented a history of contact with COVID-19-confirmed patients, health workers who were directly involved in treating the COVID-19-positive cases were included in the study. Individuals with traumatic injuries that have affected the nasopharyngeal swab collection anatomical region were excluded. Convenience sampling method was used. The sample size was calculated using the following formula:


$$\begin{array}{ll} \text{n} & ={\text{Z}}^{2}\times \frac{\text{p}\times \text{q}}{{\text{e}}^{\text{2}}}\\ & =1.96^{2}\times \frac{0.50\times 0.50}{0.07^{2}}\\ & =196 \end{array}$$

Where,

n = minimum required sample size
Z= 1.96 at 95% Confidence Interval (CI)
p = prevalence taken as 50% for maximum sample size calculation
q = 1-p
e = margin of error, 7%

The calculated sample size was 196. After adding a 10% non-response rate, the sample size was 218, However, 232 sample size was taken.

According to the WHO, patients having shortness of breath, chest pain, and loss of speech and mobility were considered to be having serious COVID-19 symptoms; fever more than 38°C (100.4°F), cough, tiredness, loss of smell or taste were considered to be having common symptoms; sore throat, headache, myalgia, diarrhoea, rashes, or discolouration of the skin, and red irritated eyes were considered to be having less common symptoms for COVID-19. Individuals presenting lower oxygen saturation (SpO_2_ <95%) with fingertip pulse oximeter were suspected to be infected.^7^ A pair of nasopharyngeal secretions swab samples was collected from each of the patients following the standard operating procedure (SOP) of the National Public Health Laboratory (NPHL) of Nepal. A swab for RT-PCR was inserted in a 3 ml viral transport media (VTM), and the other swab for SARS-CoV-2 Ag-RDT was inserted in a dedicated extraction tube provided with the kit. Samples and patient information sheets of each patient were packed separately in plastic zip-lock bags and were transported aseptically by maintaining a cold chain to the molecular laboratory of the hospital.

SARS-CoV-2 viral nucleic acid extraction was performed with spin column membrane-based purification and the amplification was performed by a multiplex RT-PCR kit for the open reading frame (ORF) lab and the N gene. An endogenous control probe labelled with a specific dye targeting the human RNase P gene was incorporated for specimen integrity, nucleic acid isolation, amplification, and detection in the reagent mixture. As the fluorescence signal changed the computer-controlled RT-PCR, the thermal cycler device drew an amplification curve such that the fluorescence produced was directly proportional to the quantity of DNA formed and fluorophore released, in each cycle continued till the end cycle for all-optical channels. The CT values obtained were those cycle numbers when the fluorescence was detectably crossed the background fluorescence, which we interpreted to determine the result as the presence/absence of SARS-CoV-2 RNA in samples. A test result was typically considered positive if amplification was observed for two or more viral targets and the positive control RNA, while it was considered negative if amplification was observed only for the positive control RNA but not for the viral targets.^8^

SARS-CoV-2 Ag-RDT tests were performed with NPHL-authorized kits using monoclonal anti-SARS-CoV-2 antibodies, targeting SARS-CoV-2 antigens such as S protein and N protein.^7,9,10^ Nasopharyngeal secretions swab samples liquefied in an extraction buffer were applied on the absorbent sample pad of the Ag-RDT kit. The analyte of interest (SARS-CoV-2 antigen in this case) if present, would bind to the colloidal gold nanoparticle-antibody conjugate at the first sight of the kit and the antigen-gold nanoparticle-antibody complex formed would flow to the other site where this complex would be captured by capture antibody to give a visible coloured band.^9^

Data were entered in Microsoft Excel 2016 and analysis was done using IBM Statistics SPSS 17.0. Point estimate and 95% CI were calculated.

## RESULTS

Among the 232 patients, COVID-19 was detected in 108 (46.55%) (40.13-52.97, 95% CI) by Ag-RDT. The mean age was 32.13±10.80 years (Table 1).

**Table 1:** Patients distribution based on age group and gender (n= 108).

Demographics		n (%)
	≤ 10	1 (0.93)
	11-20	8 (7.41)
	21-30	40 (37.04)
Age group (years)	31-40	44 (40.74)
	41-50	10 (9.23)
	51-60	3 (2.78)
	≥ 61	2 (1.85)
Gender	Male	73 (67.59)
	Female	35 (32.41)

Among the individuals with COVID-19, there were 90 (83.33%) symptomatic individuals. Most of the symptomatic COVID-19 individuals showed the presence of 2 symptoms 41 (37.96%), followed by the presence of >3 symptoms 32 (29.63%). Fever was present in 57 (52.78%), followed by dry cough in 50 (44.30%) (Table 2).

**Table 2:** Distribution of symptoms among patients with and without COVID-19 (n= 108).

Variables	n (%)
No symptom	18 (16.67)
1 symptom	17 (15.74)
2 symptoms	41 (37.96)
≥ 3 symptoms	32 (29.63)
**Symptoms**	
Anosmia	4 (3.70)
Dry cough	50 (46.30)
Fever	57 (52.78)
Headache	24 (22.22)
Myalgia	39 (36.11)
Pharyngitis	5 (4.63)
Rhinorrhea	17 (15.74)
Others	6 (5.56)

## DISCUSSION

Ever since the first case of COVID-19 caused pneumonia due to SARS-CoV-2, an enveloped RNA beta Coronavirus was reported in Wuhan, Hubei Province, China, in December 2019,^10^ the viral infection spread rapidly around the world and subsequently reached the pandemic level,^11^ as declared by the World Health Organization (WHO) on March 11, 2020.^12^ As of June 25, 2022, there were over 548 million confirmed cases of COVID-19 with more than 6 million deaths, across 228 countries and territories worldwide.^13^

In this study, the SARS-CoV-2 Ag-RDT assay showed the prevalence of COVID-19 in a tertiary care hospital to be 46.55% by Ag-RDT and 47.84% by RT-PCR. Although several studies conducted in developed nations have reported an alternative test to diagnose COVID-19, preferably with the use of SARS-CoV-2 Ag-RDT, and shed light on the diagnostic value of such a test, these studies are markedly limited in developing countries, including Nepal. Moreover, there exist differences in diagnostic accuracy among the kits, which could be attributed to several factors, including manufacturer incorporation of a well-characterized sample set for which the results are known, manufacturer inclusion of a large fraction of specimens displaying high viral loads, test conductance by highly trained personnel, who often have been involved in test development and are well aware of potential pitfalls (weak bands or other forms of ambiguous results), and variations in the quality of the nasopharyngeal swabs samples, as they were collected by different health care workers in a turbulent environment.^14,15^

This study found that COVID-19 patients aged 3140 years (39.63%) were at a higher risk of COVID-19 infection, which was discordant with the findings of several other research.^16,17^ While WHO and the Center for Disease Control and Prevention (CDC) mention a higher risk for older people to contract SARS-CoV-2 infection, a higher prevalence of COVID-19 among adults in our study could be attributable to their inability to dictate their workplace and the infeasibility of adherence to distancing and quarantine guidelines in their workplace and other communal settings.^18,19^ The mean age of COVID-19 patients (32 years) in our study was comparable with the findings from other studies.^20,21^ Several studies have discussed the different mean age groups in COVID-19 patients, ranging from 32.5 to 76 years.^21,22^ In this study, as many as twice the number of SARS-CoV-2 infections, was seen in males, which was consistent with the findings of one of the studies.^23^ The lower prevalence of SARS-CoV-2 infection in females in this study might be attributed to the adaptive immune system of females, who have higher numbers of CD4+ T cells, more robust CD8+ T cell cytotoxic activity, and increased B cell production of immunoglobulin as compared to males.^24^

The clinical course of COVID-19 is often unpredictable and is characterized by oligosymptomatic forms of the disease, presenting itself as an asymptomatic or mild case (30-60%) to a moderate or severe/critical case, which is often characterized by pneumonia (6%).^25^ In this study, 16.67% of the patients diagnosed with COVID-19 were asymptomatic. This study showed symptomatic COVID-19 patients to be frequently associated with the presence of multiple symptoms at presentation (29.63-37.96%), including fever (52.78%), dry cough (46.30%), myalgia (36.11%), headache (22.22%) and rhinorrhea (15.74%) as chief clinical complaints. The observed signs and symptoms from this study were comparable to a systematic review and meta-analysis that showed fever (88.7%), cough (57.6%), and dyspnea (45.6%) as the most prevalent symptoms in COVID-19 patients.^26^

Dependency on the viral load concentration is a limitation associated with this serological method, as the viral load in patients gradually changes with the severity or less severity of the disease.

## CONCLUSIONS

The prevalence of COVID-19 among hospitalized individuals in this study was higher than in previous studies conducted in similar settings.
